# Clinical characteristics and genetic profiles of 174 patients with X-linked agammaglobulinemia: Report from Shanghai, China (2000–2015)

**DOI:** 10.1097/MD.0000000000004544

**Published:** 2016-08-12

**Authors:** Xia-Fang Chen, Wei-Fan Wang, Yi-Dan Zhang, Wei Zhao, Jing Wu, Tong-Xin Chen

**Affiliations:** a Department of Allergy and Immunology, Shanghai Children's Medical Center; b Division of Immunology, Institute of Pediatric Translational Medicine, Shanghai Jiao Tong University School of Medicine, Shanghai; c Department of Internal Medicine, The Affiliated Hospital to Changchun University of Chinese Medicine, Changchun, China; d Division of Allergy and Immunology, Department of Pediatrics, Virginia Commonwealth University, Richmond, VA.

**Keywords:** Bruton tyrosine kinase, Chinese, humoral immunodeficiency, X-linked agammaglobulinemia

## Abstract

X-linked agammaglobulinemia (XLA) is a humoral primary immunodeficiency. XLA patients typically present with very low numbers of peripheral B cells and a profound deficiency of all immunoglobulin isotypes. Most XLA patients carry mutations in Bruton tyrosine kinase (*BTK*) gene.

The genetic background and clinical features of 174 Chinese patients with XLA were investigated. The relationship between specific *BTK* gene mutations and severity of clinical manifestations was also examined. Mutations were graded from mild to severe based on structural and functional prediction through bioinformatics analysis.

One hundred twenty-seven mutations were identified in 142 patients from 124 families, including 45 novel mutations and 82 recurrent mutations that were distributed over the entire *BTK* gene sequence. Variation in phenotypes was observed, and there was a tendency of association between genotype and age of disease onset.

This report constitutes the largest group of patients with BTK mutations in China. A genotype–phenotype correlation was observed in this study. Early diagnosis of congenital agammaglobulinemia should be based on clinical symptoms, family history, and molecular analysis of the *BTK* gene.

## 1 Introduction

X-linked agammaglobulinemia (XLA, MIM# 300755) is a primary immunodeficiency due to human B-lymphocyte development disorder, thus resulting in antibody deficiency and recurrent bacterial infection. ^[1]^ The abnormal gene in XLA maps to q22 on the long arm of the X chromosome and encodes the B-cell protein tyrosine kinase BTK (Bruton tyrosine kinase). ^[2]^ BTK is composed of 5 distinct structural domains, namely, Pleckstrin homology (PH), Tec homology (TH), Src homology (SH3), SH2, and catalytic kinase (SH1) domains. [^3^ ^4^ ^5^ ^6^ ^7^] The human *BTK* gene is located in the Xq22 region of the X chromosome. It encompass 37.5 kb including 19 exons. Eighteen of them encode protein. A cluster of transcriptional start sites has been identified upstream of exon 1. Both in vivo and in vitro studies have proved that BTK protein is essential for the survival, cell cycle progression, and proliferation of B cells in response to surface Ag receptor stimulation. ^[8]^ The defective BTK in XLA impairs early B-cell development, resulting in a marked reduction of mature B cells in peripheral blood. Reports from different countries and ethnic groups have demonstrated that approximately 90% of males with presumed XLA have mutations in *BTK*. Online BTK database contain 1155 entries compiled from 974 unrelated families with 602 unique molecular events, indicating majority of *BTK* mutations being random. Results from several large cohort studies added valuable knowledge to our understanding of the spectrum of clinical features of XLA. [^9^ ^10^ ^11^ ^12^ ^13^] Two recent reports suggest the presence of genotype–phenotype correlations[ ^14^ ^15^] ; this is in contrast to earlier publications. ^[16]^ The genetic and epidemiological characteristics of XLA remain largely unexplored in the mainland of China. The current study provides clinical presentation and *BTK* mutation profile of 174 Chinese XLA patients.

## 2 Materials and methods

### 2.1 Patients

One hundred seventy-four patients were included for this retrospective analysis. They were evaluated in the immunodeficiency clinic at the Shanghai Jiao Tong University School of Medicine from 2000 to 2015. The initial diagnosis of XLA of majority of patients was made in our clinic. A few patients were referred for genetic counseling and molecular diagnostic analysis after clinical diagnosis made in other areas of China. The age of diagnosis is defined as the age at first clinical visit. The diagnosis of XLA was made based on the criteria of Pan-American Group for Immunodeficiency (PAGID, 1999) and European Society for Immunodeficiencies for primary immunodeficiency diseases (ESID). ^[17]^

#### 2.1.1 Definitive diagnosis

Male patient with less than 2% CD19 B cells and at least one of the following:

1. Mutation in Btk.
2. Absent Btk mRNA on Northern blot analysis of neutrophils or monocytes.
3. Absent Btk protein in monocytes or platelets.
4. Maternal cousins, uncles, or nephews with less than 2% CD19 B cells.

#### 2.1.2 Probable diagnosis

Male patient with less than 2% CD19 B cells with all of the following:

1. Onset of recurrent bacterial infections in the first 5 years of life.
2. Serum IgG, IgM, and IgA more than 2 SD below normal range for age.
3. Absent isohemagglutinins and/or poor response to vaccines.
4. Other causes of hypogammaglobulinemia have been excluded.

#### 2.1.3 Possible diagnosis

Male patient with less than 2% CD191 B cells in whom other causes of hypogammaglobulinemia have been excluded and with at least one of the following:

1. Onset of recurrent bacterial infections in the first 5 years of life.
2. Serum IgG, IgM, and IgA more than 2 SD below normal range for age.
3. Absent isohemagglutinins.

The informed consent for genetic testing was obtained from parents. This study was approved by Shanghai Children's Medical Center Investigation Committee.

### 2.2 *BTK* mutation detection

#### 2.2.1 *BTK* gene analysis

Genomic DNA of study patients was extracted from blood leukocytes according to standard protocols. The *BTK* gene was amplified from cDNA by using a set of specific primers with a single annealing temperature and the same conditions for all the segments as previously described.[ ^18^ ^19^] Mutations were detected by sequencing from the opposite direction with the National Center for Biotechnology Information program Basic Local Alignment Search Tool (http://www.ncbi.nlm.nih.gov/BLAST/).

### 2.3 Genotype–phenotype correlation

Mutations were classified into “severe” or “less severe” as previously described.[ ^14^ ^15^ ^20^] Frameshift and nonsense mutations leading to protein truncation were considered as severe mutations. We used Polymorphism Phenotyping v2 (PolyPhen-2), ^[21]^ Sorting Intolerant From Tolerant (SIFT), ^[22]^ and MutationTaster ^[23]^ to predict the severity of missense mutation, whether an amino acid substitution affects protein function. Disease severity was gauged by the types of infections before the diagnosis.

### 2.4 Statistical analysis

The demographics, immunological data, and clinical characteristics were depicted by descriptive statistics. Chi-square tests were performed to test for significant differences between genotypes and phenotypes. Student unpaired *t* test was used to compare other variables between groups. SPSS 17.0 statistical software was used to perform analyses.

## 3 Results

### 3.1 Demographic data

The 174 XLA patients were from 22 provinces and municipalities throughout China. Among them, 110 patients (63.22%) came from 6 provinces and 1 municipality in East China, whereas the other 64 patients (36.78%) were from other 15 provinces. One hundred forty patients (80.46%) were diagnosed from 2007 to 2015, whereas only 20 patients (11.49%) were diagnosed from 2001 to 2007 (Table 1 ).

**Table 1:**
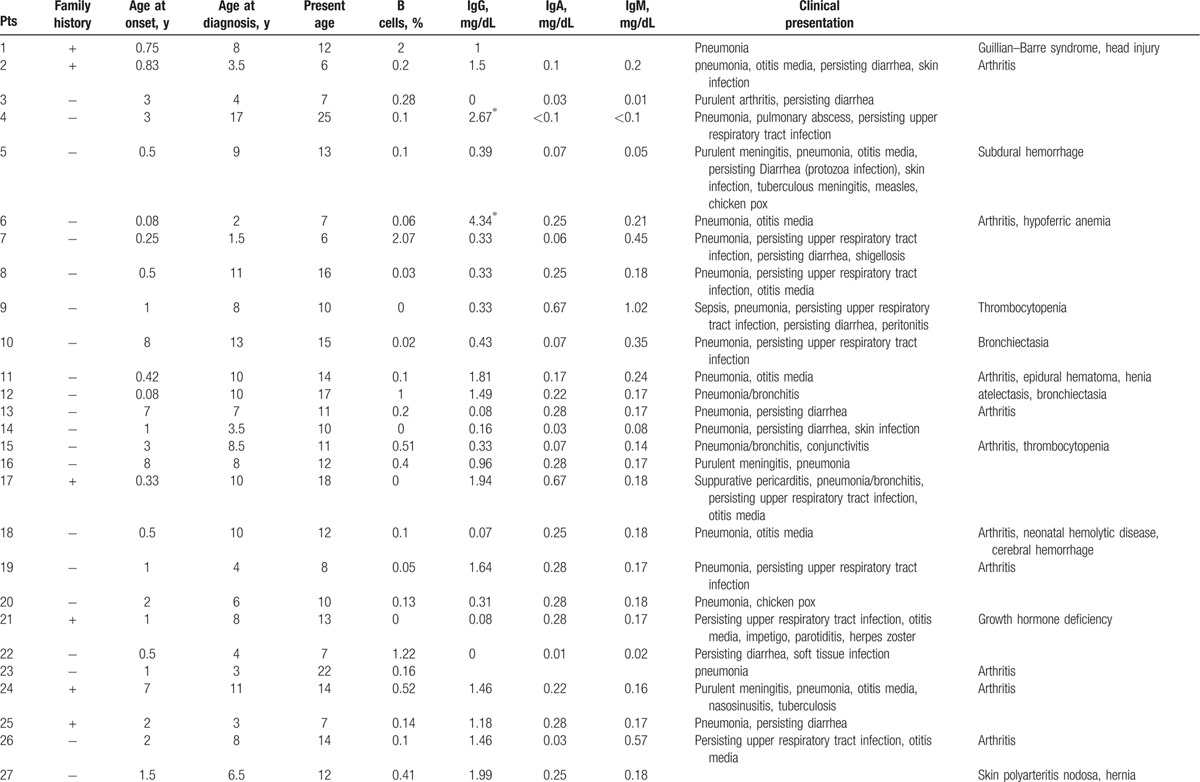
Characteristics features of 174 XLA patients.

**Table 1 (Continued):**
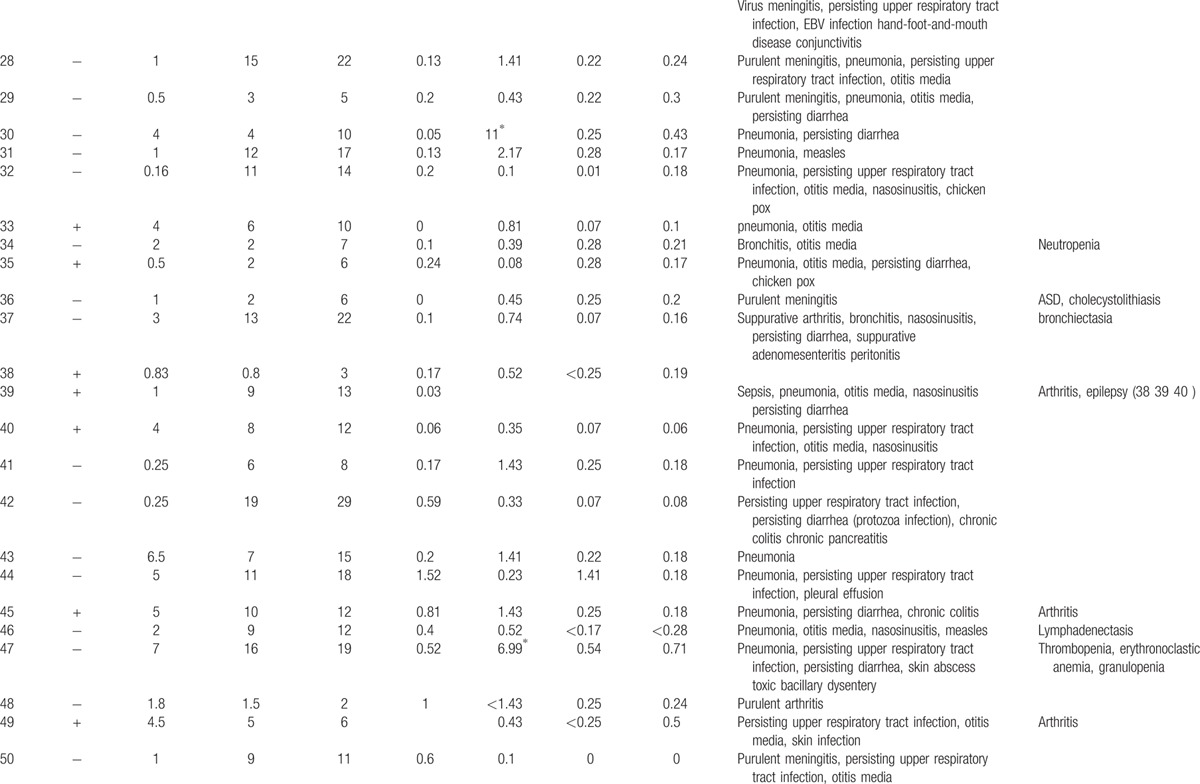
Characteristics features of 174 XLA patients.

**Table 1 (Continued):**
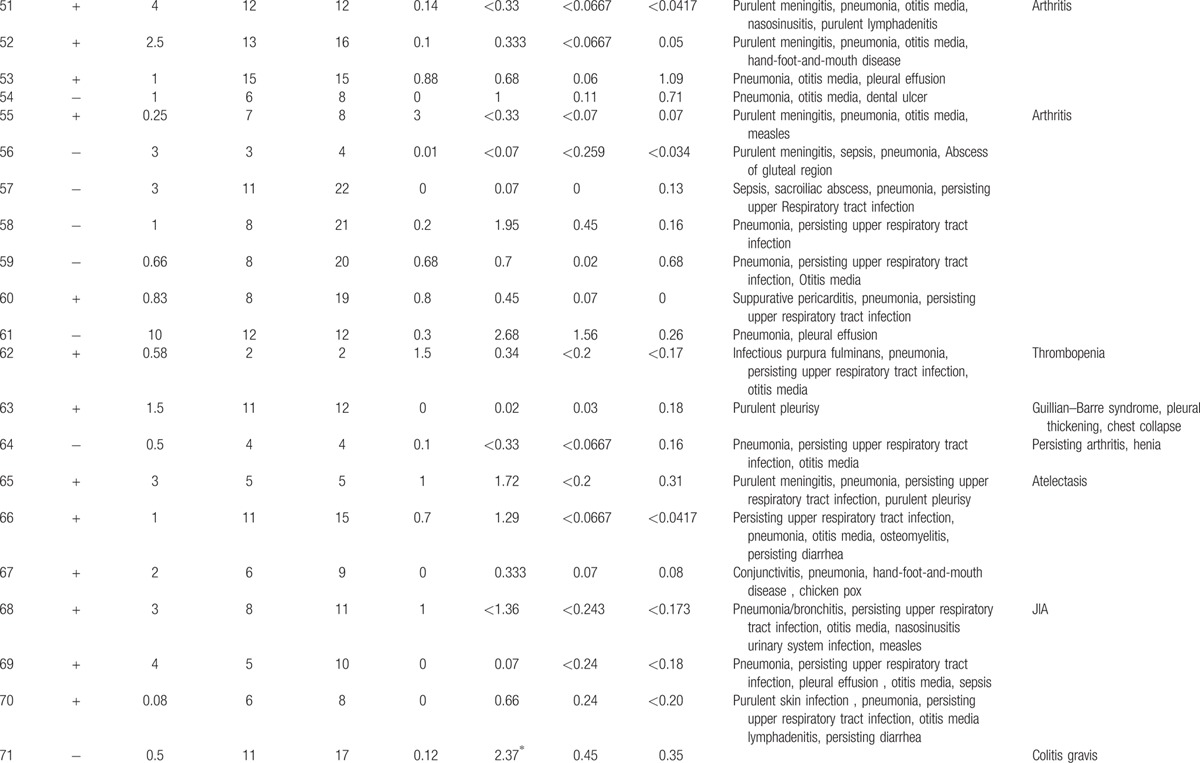
Characteristics features of 174 XLA patients.

**Table 1 (Continued):**
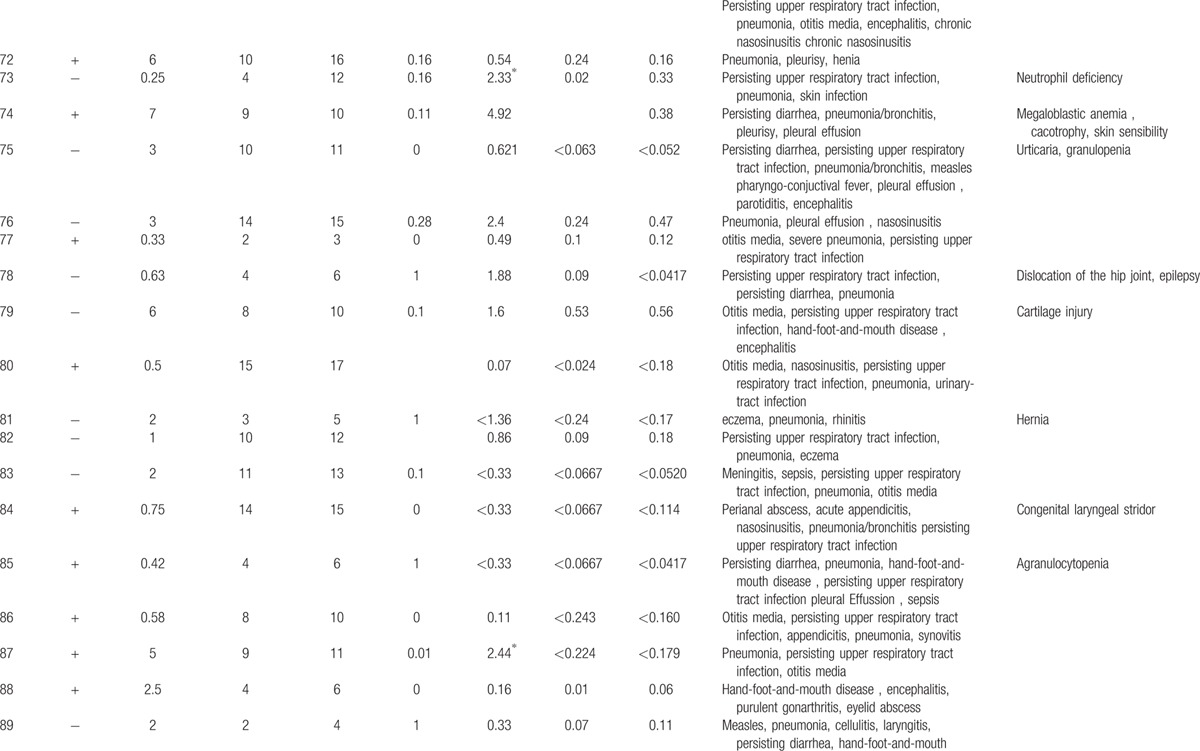
Characteristics features of 174 XLA patients.

**Table 1 (Continued):**
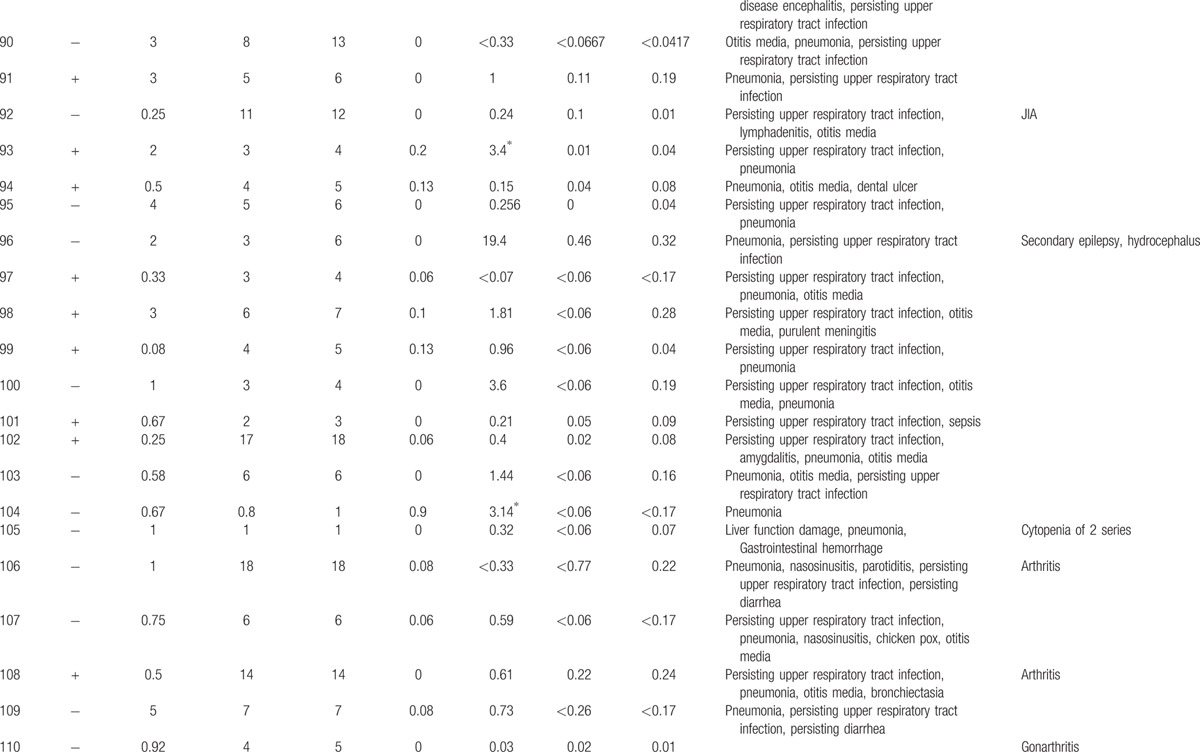
Characteristics features of 174 XLA patients.

**Table 1 (Continued):**
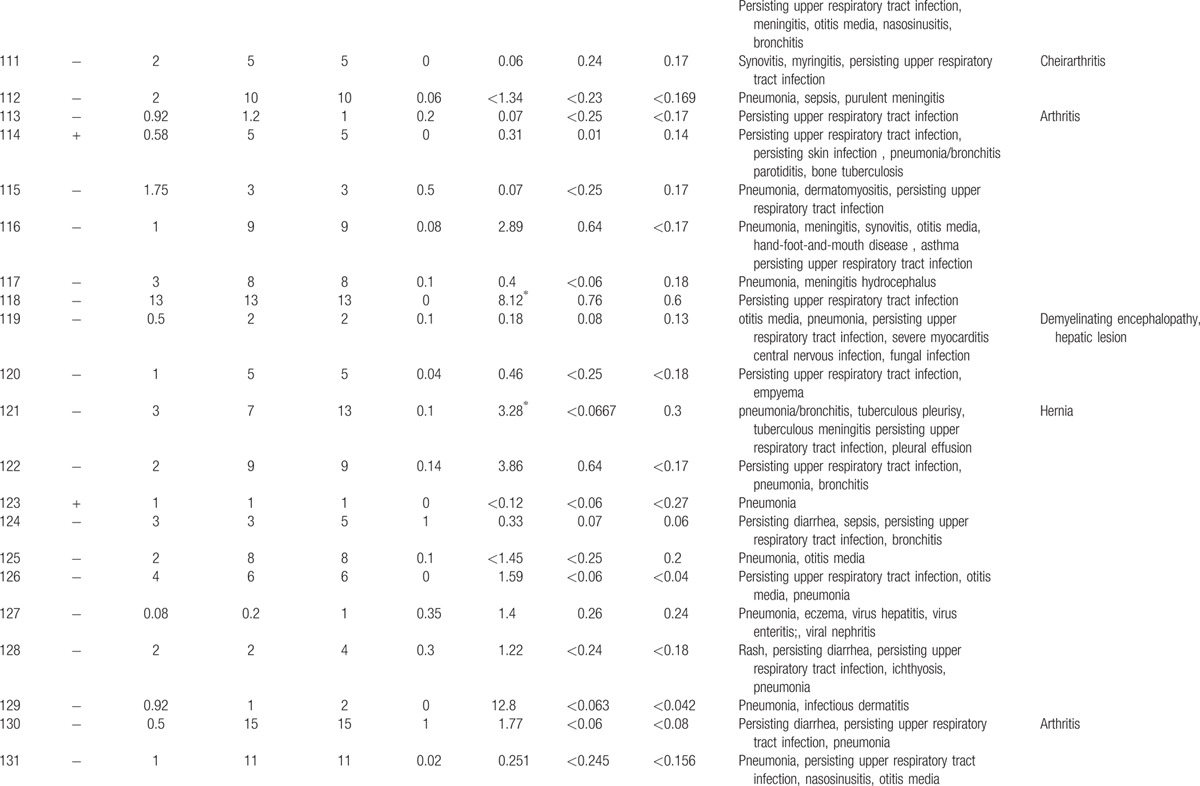
Characteristics features of 174 XLA patients.

**Table 1 (Continued):**
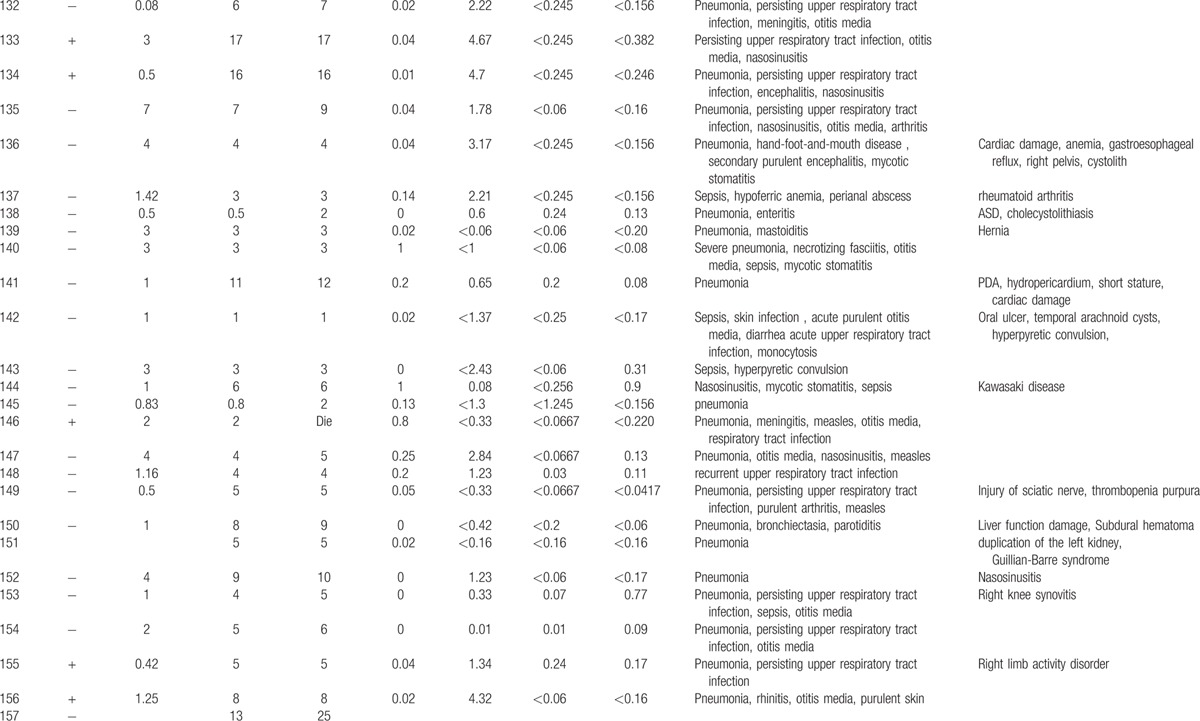
Characteristics features of 174 XLA patients.

**Table 1 (Continued):**
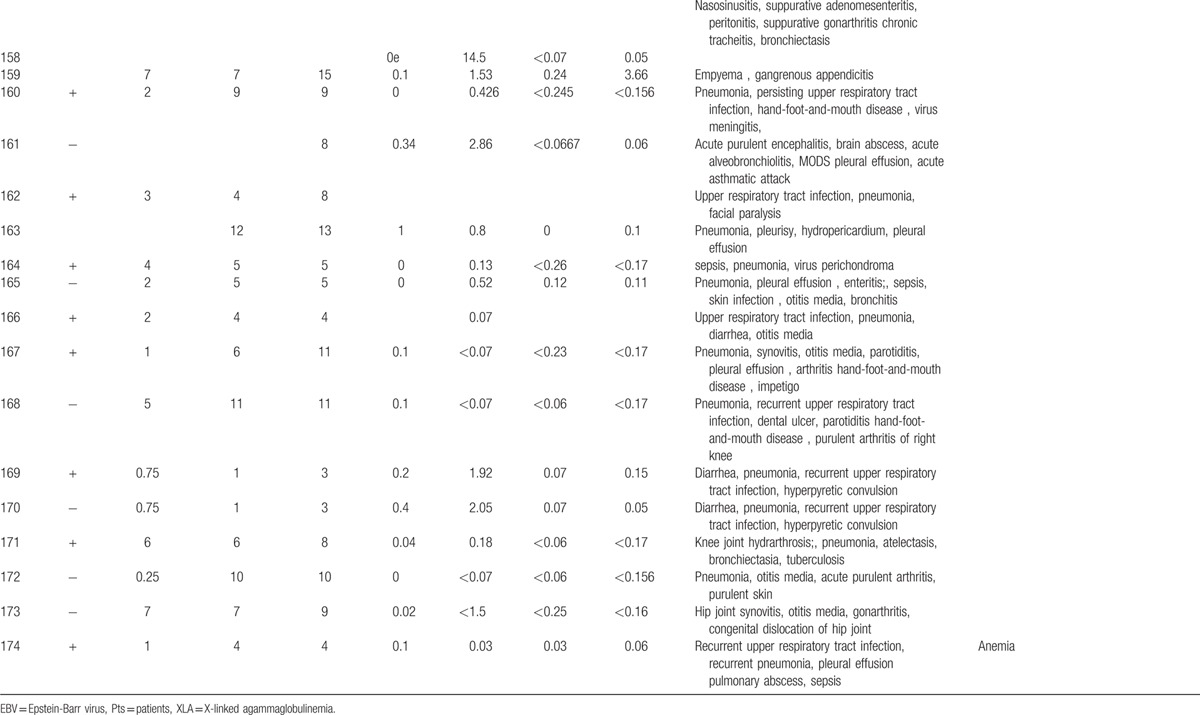
Characteristics features of 174 XLA patients.

The 174 patients included 127 cases with definite diagnosis, 31 cases with probable diagnosis, 12 cases with possible diagnosis, and 3 cases with clinical suspicion.

The age of onset of those patients was 2.15 ± 2.16 years with median of 1. One hundred thirty-five patients (77.59%) had onset before 3 years of age. Only 1 patient manifested symptoms at 13 years of age.

The average age of diagnosis was 7.09 ± 3.98 years, with range from 0.17 to 19. Eighty-seven patients (50.58%) were diagnosed during the first 6 years. However, only in 5 patients (2.91%), the diagnosis was made before 1 year of age.

**Figure 1:**
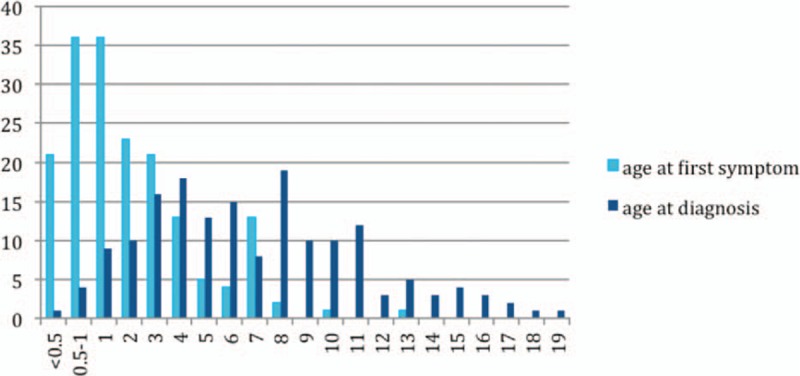
Distribution of the age of disease onset and diagnosis in patients with XLA.

Family histories were available from 170 of the 174 patients. Fifty-nine patients (34.71%) had a positive family history (i.e., early male abortion within 3 generations in the family), whereas the other 111 patients (65.29%) had no family history of immunodeficiency. Patients 38, 39, and 40 were cousins. Patients 169 and 170 were twins (Fig. 1).

### 3.2 The level of serum immunoglobulin and the percentage of CD19+ B Cells

Peripheral CD19 + cells were tested in 168 of 174 patients at the time of diagnosis. The percentage of CD19+B cells was less than 2% in 165 patients, 2% in 1 patient (P1), and 3% in another (P7). Serum IgG levels were tested in 171 patients with IgG <2 g/L in 142 patients. Among the 29 patients with IgG >2 g/L, 11 received IVIG 1 month before the testing was performed. However, CD19+B cells were below 2% in all 29 patients.

Serum IgA was measured in 168 of 174 XLA patients, and IgM was measured in 169. Compared with the healthy counterparts, ^[24]^ 166 patients had IgA level below low limit of normal, ranging from 0.00 to 0.76 g/L. Meanwhile, 166 (98.22%) patients showed IgM level from 0.01 to 0.71 g/L.

### 3.3 Related infections and accompanied symptoms

Clinical manifestations of infection were identified in 173 patients before XLA was diagnosed, predominately respiratory tract infection, which accounted for 94.25% (164 patients) of the infections.

Lower respiratory tract infection (bronchitis/pneumonia) were found in 134 cases (77.01%), whereas otitis media was found in 70 cases (40.23%), persisting diarrhea in 33 cases (18.97%), nasosinusitis in 23cases (13.22%), and skin infections in 14 cases (8.05%).

Fifty-eight patients (33.33%) suffered severe infections, such as central nervous system infection (34 cases), sepsis (15 cases), suppurative arthritis (6 cases), suppurative pericarditis (2 cases), bone tuberculosis (1 case), osteomyelitis (1 case), and purpura fulminans (1 case).

Upper respiratory tract infection and otitis media were the most common symptoms in this patient population. Individually, upper respiratory tract infection occurred 20 times a year, whereas otitis media occurred 10 times in 1 patient.

As illustrated in Table 2 , all 174 patients had other complications such as arthritis (32 cases, 18.39%), neutropenia or thrombocytopenia (9 cases, 5.17%), polyarteritis nodosa (P27, 0.57%), growth hormone deficiency (P21, P141, 1.15%), megaloblastic anemia (P74, 0.57%), and infection after vaccination of poliovaccine (P31, 0.57%). The rate and distribution of related infections and accompanied symptoms in XLA patients were showed in Table 3.

**Table 2:**
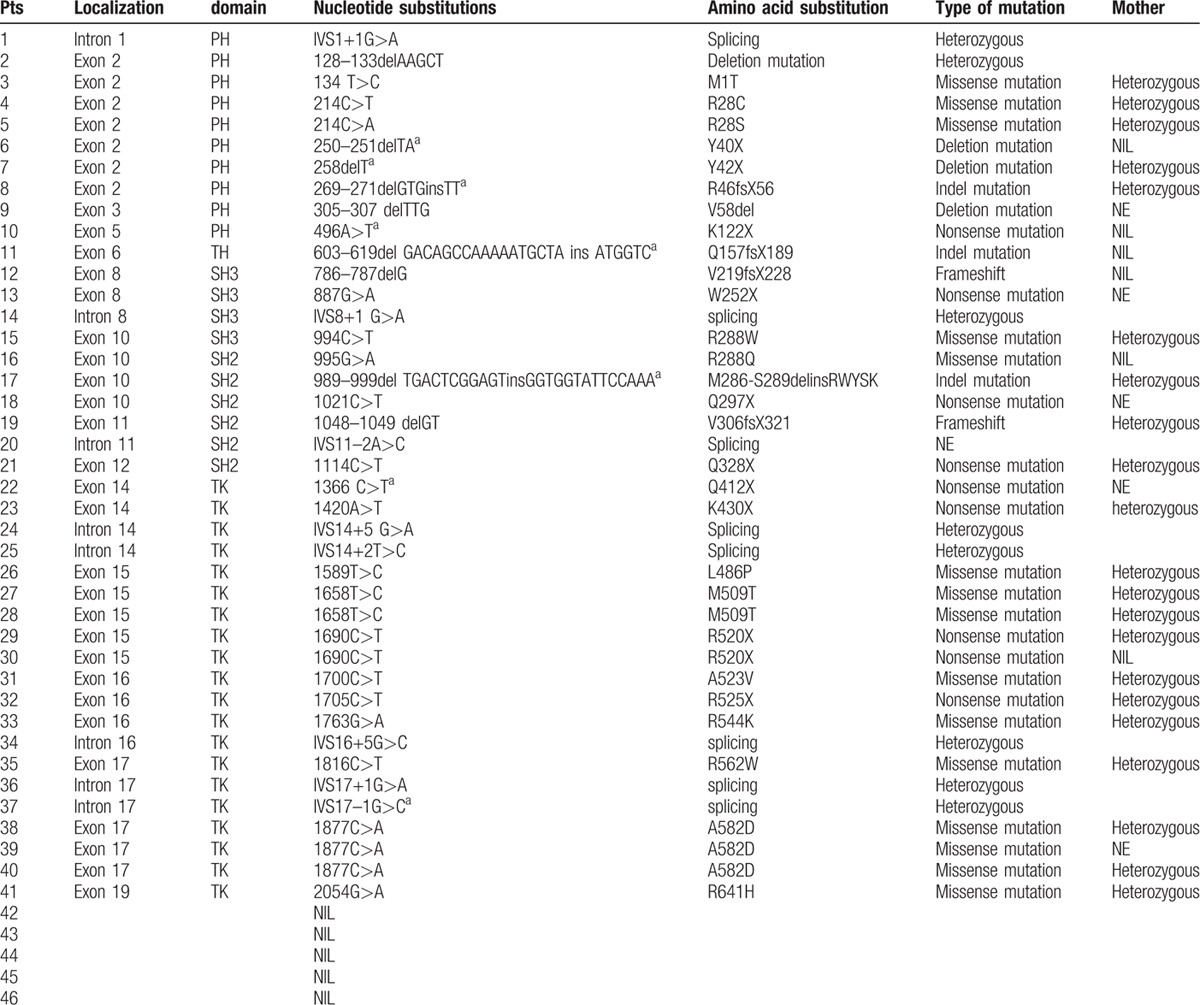
Btk mutation analysis in 142 children with XLA in this study.

**Table 2 (Continued):**
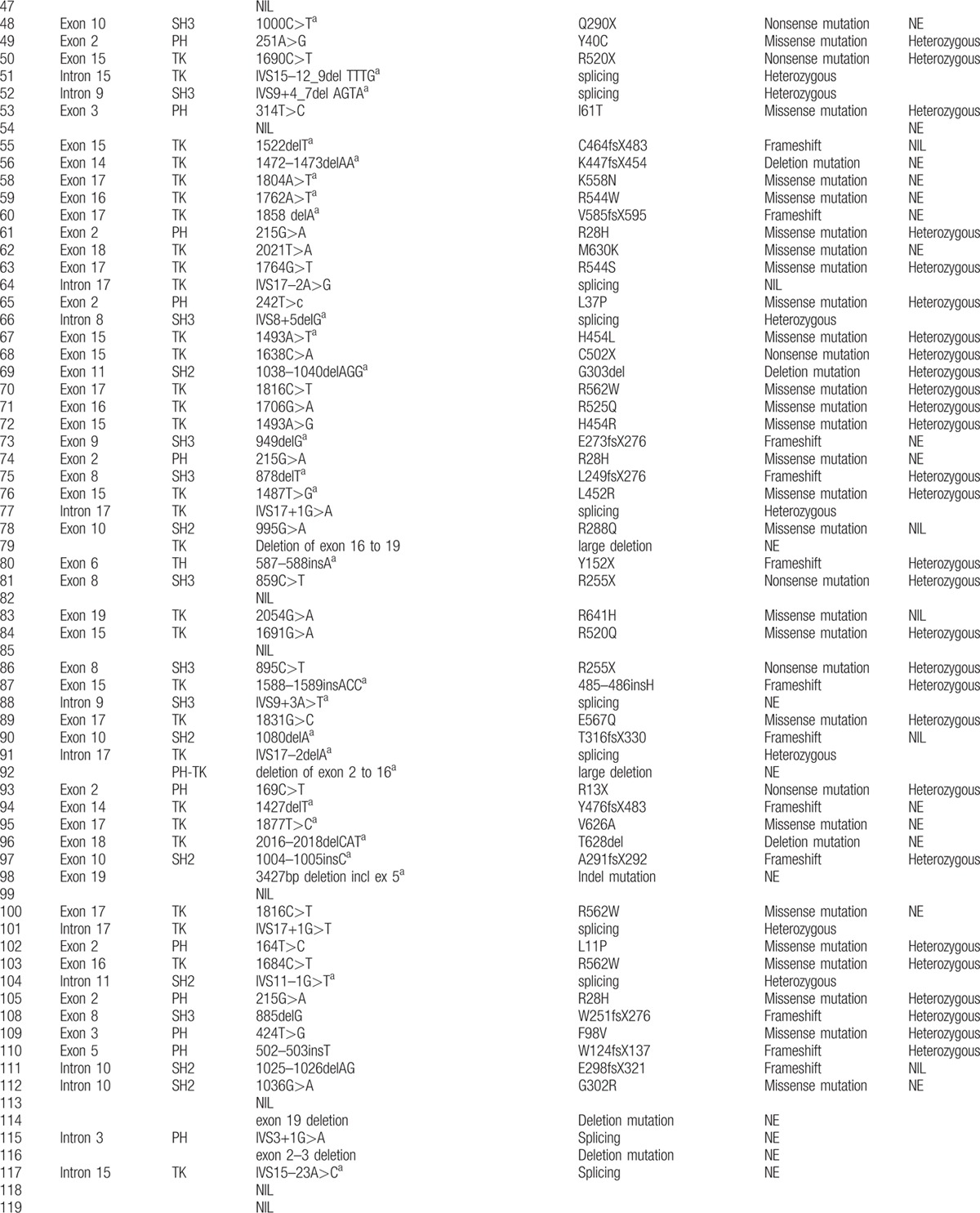
Btk mutation analysis in 142 children with XLA in this study.

**Table 2 (Continued):**
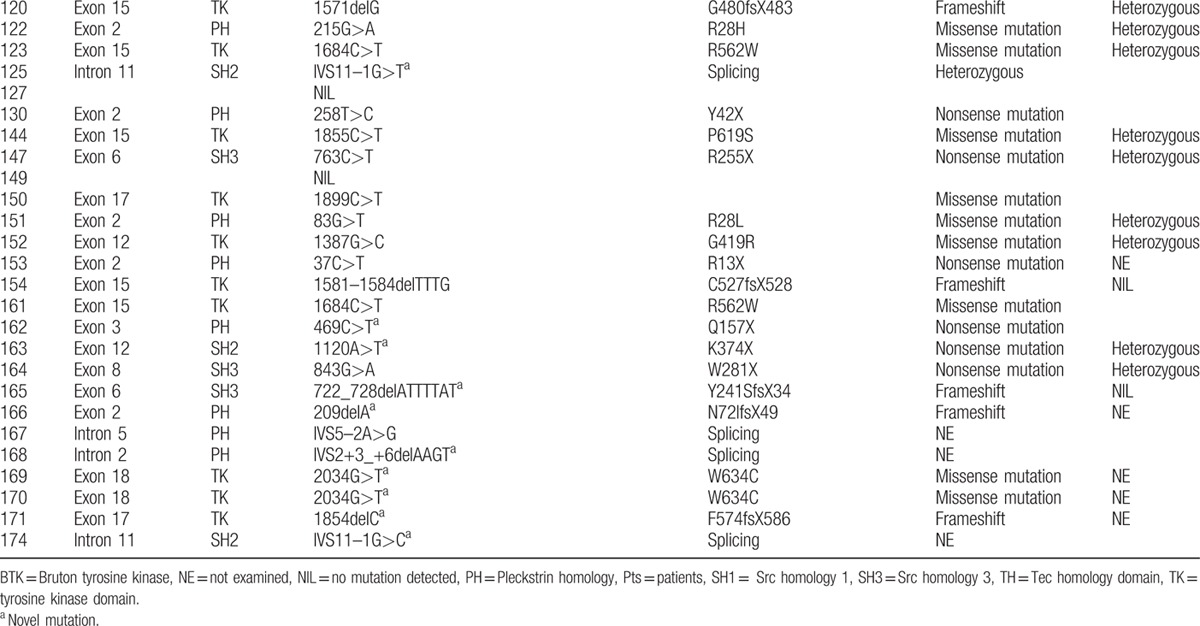
Btk mutation analysis in 142 children with XLA in this study.

**Table 3:**
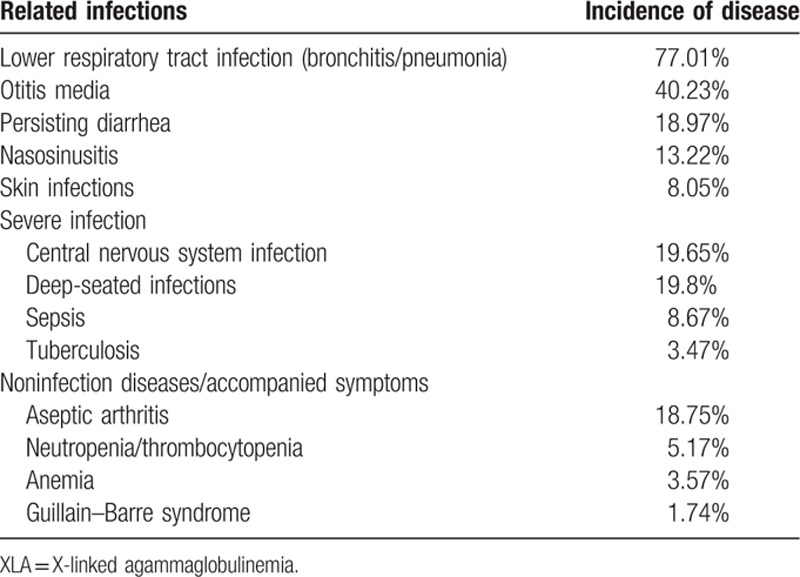
The frequency data of related infections and accompanied symptoms in the XLA patients.

### 3.4 Correlation analysis of clinical characteristics

There is significant difference in number of cases diagnosed before and after year 2007. The diagnosis of 154 patients was made after 2007, with the onset age of 2.21 ± 2.18 years, whereas only 20 patients were diagnosed before 2007, with the onset age of 1.76 ± 1.91 years. The age of diagnosis was not statistically different (*t* = 0.804, *P* = 0.422). But the interval between onset of symptoms and XLA diagnosis was significantly different in those 2 groups, with 4.36 ± 3.13 and 7.69 ± 3.32 years, respectively, for after and before 2007 groups (*t* = −2.840, *P* = 0.005).

The average age of XLA diagnosis in patients with nasosinusitis as complication was 10.63 ± 3.97 years, which is significantly later than those without nasosinusitis (6.41 ± 4.04 years; *t* = 4.653, *P* < 0.0001).

No significant correlations were noticed between the severity of infections, family history, or region of the disease. Twenty-two of the 60 (36.67%) patients with positive family history were found to have severe infections, and only 36 (33.33%) in patients without family history (*χ* = 0.190, *P* = 0.753).

### 3.5 BTK mutation analysis

**Figure 2:**
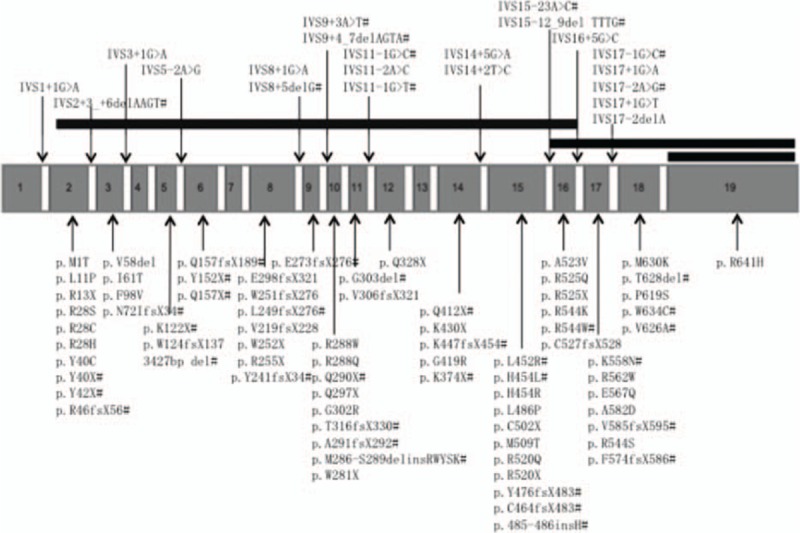
Schematic representation of BTK mutations detected in the present study.

The *BTK* gene analysis was performed in 142 of 174 XLA patients. One hundred twenty-seven patients, coming from 124 individual families, were found to have *BTK* gene mutation, including 45 novel mutations (Table 2 ). Forty-eight patients were found to have missense mutations (38.10%). Frameshift due to insertions or deletions accounted for 23.02% (29 patients). Splicing mutations were identified in 23 patients (18.25%), nonsense mutations in 21 (16.67%), and large deletions in 5 (3.97%). The locations of mutations were scattered throughout the *BTK* gene, with TK domain being most frequent (n = 64, 50.79%), followed by the PH domain (n = 28, 22.22%), the SH2 domain (n = 16, 12.70%), the SH3 domain (n = 15, 11.90%), and the TH domain (n = 3, 2.38%) (Fig. 2). We also identified 1 large deletion between PH domain and TK domain and another large deletion affecting region between exon 16 and exon 19 (patients 33 and 40, respectively). Forty-five new mutations were identified when comparing with the online BTK database (http://structure.bmc.lu.se/idbase/BTKbase/), including frameshift (20), missense (7), splicing (11), nonsense (5) mutations, and large deletions (2).

### 3.6 BTK genotype–phenotype correlation

The age of onset of the 93 patients with severe genotype mutation was 1.91 ± 1.84 years, which was significantly earlier than that of 27 patients with mild genotype mutation (2.83 ± 2.32 years; *t* = −2.180, *P* = 0.031). The average age of diagnosis in severe genotype patients was 6.34 ± 3.82 years, which was significantly earlier than that of mild genotype (*t* = −2.751, *P* = 0.007).

Thirty-three of the 50 (66.0%) patients with positive family history had severe genotype. However, this rate is much higher (60 of 72, 83.33%) in patients without family history (*χ* = 4.893, *P* = 0.032). No correlations were found regarding the severity of infections, the severity of genotype, or arthritis as complication. Thirty-two of the 42 (76.19%) patients with severe infections had severe genotype, and those with mild infections was 63 of 82 (76.82%), not statistically different (*χ* = 0.006, *P* = 1.000).

Twenty-one of the 26 (80.76%) patients with arthritis as complication had severe genotype, and the rate in those without arthritis was 65 of 88 (73.86%), which was not statistically significant (*χ* = 0.517, *P* = 0.607).

## 4 Discussion

X-linked agammaglobulinemia accounts for 6% to 11% of primary immunodeficiency (PID). The reported incidence of XLA varies in different countries. It is 1/200,000 live births in Switzerland, 1/10,000,000 to 1/20,000,000 live births in Spain, 1/100,000 to 1/285,000 live births in Norway and 1/379,000 live births in the United States.[ ^11^ ^26^ ^27^ ^28^] It is difficult to accurately access the incidence of XLA in mainland of China due to the lack of national or local PID registration. Based on the population census of 16.87 million new birth populations in 2014, using the incidence of Norway, the new cases of XLA would be above 80 annually, and the cumulative cases below 14 years of age should be above 1000. In our center, the number of XLA cased among total diagnosed PID is higher than that of other countries, that is, 174 diagnosed in 14 years. It is partially due to simple tested required for making the diagnosis and longer survival of XLA patients. However, further investigations are still needed to delineate the mechanism. Since we are one of the 2 major medical centers in the entire mainland of China managing those patients, our cohort is 174, much lower than the estimated 1000 cases, we predict that there are still significant number of patients who are not diagnosed. The fact that much more XLA cases were diagnosed after 2007 suggests physicians have increased index of suspicion on immunodeficiency patients in China.

There are 31 provinces and municipalities in mainland China. We noticed a skewed geographic distribution of XLA patients. Among the 174 XLA patients reported here of 22 provinces and municipalities, most of them were from the 6 provinces and 1 municipality in East China. It might be due to the fact that Shanghai is the center of the district with convenient transportation and advanced medical technology.

Infection is the primary presentation of XLA patients. In our cohort, all but patients 38 and 130 had significant infection before the diagnosis. Similar to other countries, respiratory tract infection is the most common infection among which pneumonia constitutes the largest in our patient group. Otitis media and recurrent diarrhea are also very common, whereas nasosinusitis and cutaneous infection are less frequent. In terms of severe infection, infection of the central nervous system is the most common, whereas sepsis and suppurative arthritis are relatively rare. Examples are osteomyelitis (patient 66) and hepatitis (patient 127), and also tubercular meningitis.

The cohort described here demonstrated higher rates of lower respiratory tract infection (bronchitis/pneumonia, 79.3%), recurrent upper respiratory tract infection, and otitis media, which were different from those from other countries. In Netherlands, 100% of the 15 XLA patients suffered from pneumonia. ^[29]^ In USA, 62% of the patients suffered pneumonia and 70% of those suffered from otitis media. ^[11]^ In the current study, the incidence of otitis media was 41.5%. Data suggest a higher risk of XLA in patients with pneumonia, recurrent upper respiratory tract infection, otitis media, and chronic nasosinusitis.

On the contrary, the incidence of noninfection arthritis was 18.75% in our patient population, similar to what Wang et al observed. ^[30]^ However, such numbers are very different from what was reported from other countries. Of note, the onset of arthritis in XLA patients was 6 years of age in average, with youngest at 1 year of age. This is significantly earlier than that of juvenile idiopathic arthritis (JIA), indicating high risk of septic joint infection in XLA patients.

Cases of poliomyelitis after polio vaccination had been reported in XLA patients.[ ^11^ ^12^] In our cohort, 1 patient received the attenuated polio vaccination and presented with flaccid paralysis 2 months later. Due to the lack of viral serotyping, it is not clear if the infection was caused by polio vaccine or natural wild-type polio virus.

Polyarteritis nodosa with cat eye syndrome chromosome region, candidate 1 gene mutation can display low B cells and immunodeficiency. One of our patients was diagnosed with polyarteritis nodosa. This patient was referred to us due to 5 years history of recurrent respiratory tract infection and bilateral intermittent red rash of legs for 6 months. The diagnosis of polyarteritis nodosa was made based on history and physical examination. The laboratory examination showed low B cells and immunodeficiency, but negative rheumatoid factors, antinuclear antibody, and normal ESR. *BTK* gene sequencing confirmed BTK mutation.

**Figure 3:**
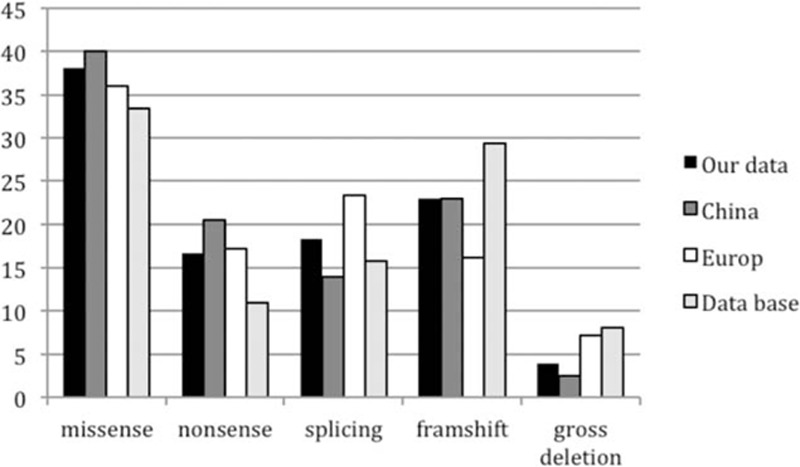
Distribution of BTK mutation types in 4 large cohorts.

The 82 (64.29%) patients showed the recurrent mutations as previously described (BTKbase; http://bioinf.uta.fi/BTKbase). The spectrum of mutations are very similar to a previous cohort study of eastern and central Europe, currently the largest reported group of *BTK* mutation in Europe. ^[31]^ It comprised of 122 patients with 53.1% recurrent mutations. Previous studies in Chinese cases of XLA in the literature reported 74 mutations from 74 patients (Table 4 ). [^32^ ^33^ ^34^ ^35^ ^36^ ^37^ ^38^] When combining our data with published Chinese cases, we found that the collective data are comparable with the large European data and the BTK database (Fig. 3), with missense being most common, followed by frameshift, splicing, and nonsense mutation, whereas the gross deletions being the rarest. In addition, the frequency of nonsense mutation in our group was similar to the European data (16.7% and 17.2%, respectively) that is higher than that in the BTK database (11.53%), whereas previous reported Chinese group had the highest frequency (21.08%). The gross deletion rate was very low in reported Chinese data (1.22%) compared with the European data and BTK database. However, the overall pattern of mutations from our cohort and previously reported Chinese data is consistent with what was found in BTK database.

**Table 4:**
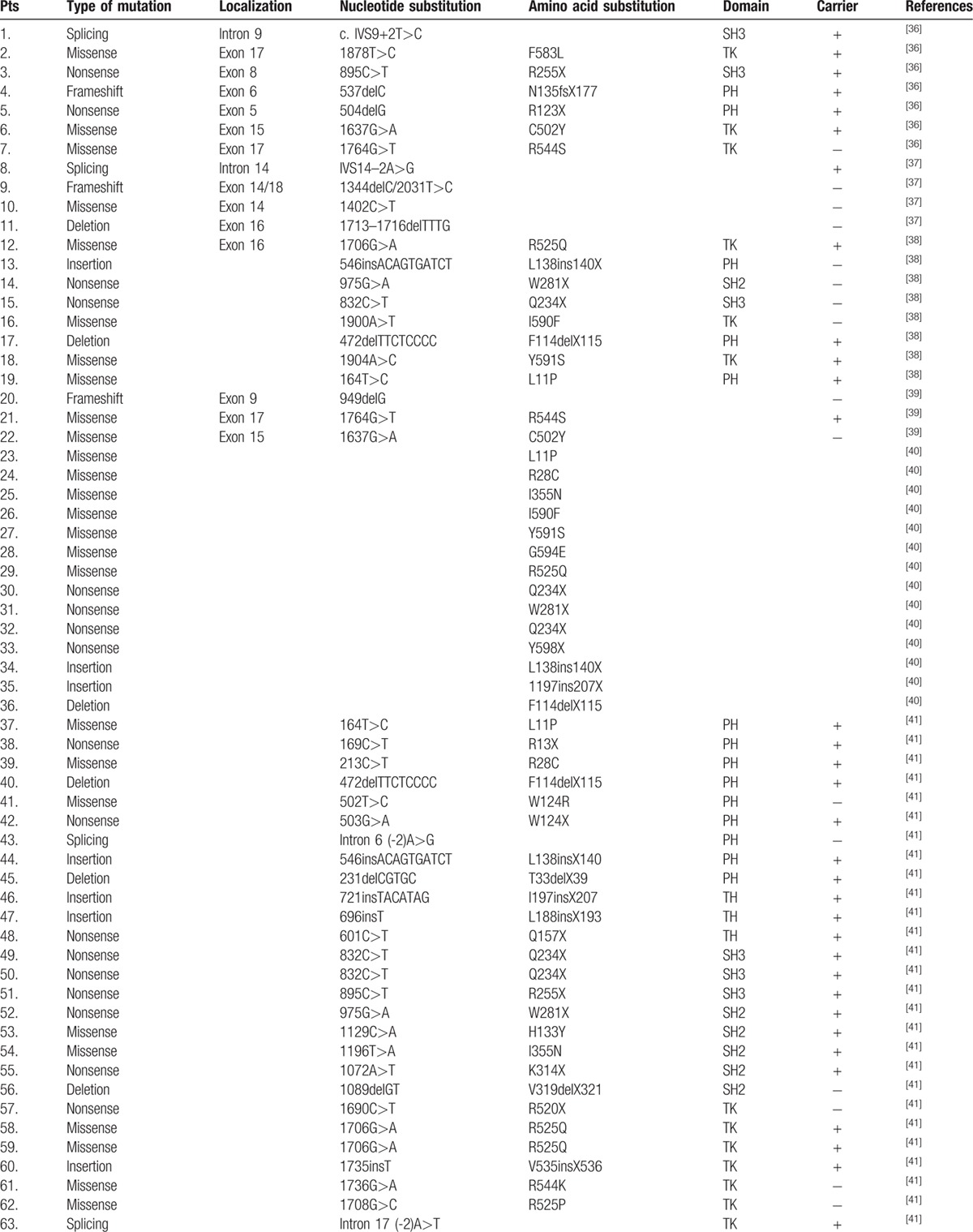
Seventy-four patients with BTK mutations published in Chinese version.

**Table 4 (Continued):**
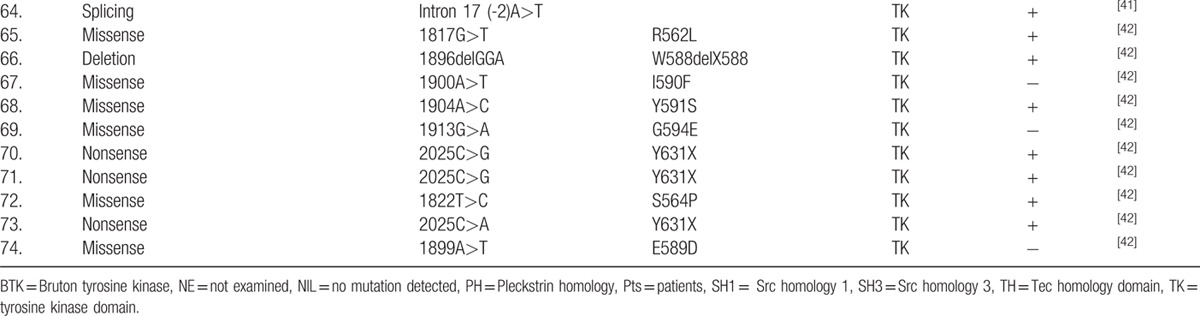
Seventy-four patients with BTK mutations published in Chinese version.

The most common mutation sites in our study were R520 (1 R520C, 3 R520X) and R525 (1 R52Q, 2 R525X), which are also the recurrent mutation sites in BTK in arginine-coding CpG dinucleotides. These sites contain the sequence of purine-CpGpyrimidine, which is the single most mutable tetranucleotide.[ ^16^ ^39^] Several studies have described mutations at this codon. [^40^ ^41^ ^42^] BTK database showed 20 of the mutations resulting in R520X and 17 of them leading to R520. BTK database also lists 4 additional mutations in codon R525 in 23 patients. The defined “hot spot” suggests an important functional element associated with the wild-type arginine in the BTK domain.

The second most common recurrent mutation site in our study was R28 (3 R28H, 1 R28C). It was reported that different amino substitution in this site resulted in varying degree of impairment and dysfunction. The functional mutation in R28C was shown to be less severe than R28H. ^[43]^ As reported previously, the effect of mutation of R28C was milder on XID (sex-linked immunodeficiency) mice compared with that on XLA patients. ^[44]^ This was consistent with the finding in our study that the patients with R28C mutation had milder clinical features.

A582D was the most frequently identified novel mutation in this study and was found in 3 patients (cousins) from 1 family. The other 2 substitutions of Ala to Asp in BTK database are A508D and A607D. The mutation A508D introduces a charged residue in the hydrophobic core of the domain and, therefore, is likely to alter its conformation. Polar residues are not allowed to reside in a protein core unless the charge can be neutralized. Another compensatory mutation would be required to tolerate the A508D mutation. ^[45]^ A582 site was reported to be changed to Val in BTK database. The side chain of Trp-563 is sandwiched between Arg-562 and Ala-582. Mutation of either of the surrounding residues could thus interfere with it sterically. ^[46]^. Serine does not have the bidentate structure of valine. We speculate that A582D may lead to alteration of BTK structure.

Not all patients with the early onset of infections, profound hypogammaglobulinemia, and markedly reduced or absent B cells are found to have BTK mutations. Mutations in pre-B-cell receptor can disrupt B-cell development. ^[47]^ In the present study, 15 patients were diagnosed as XLA, but no *BTK* mutation was detected. Conley et al stated that BTK mutation accounts for about 85% of patients with defects in early B-cell development. The remaining patients have defects that are heterogeneous. About one-third have mutations in μ-heavy chain; a small number have defects in λ5, Igα, or B-cell linker. In 5% to 10% of patients, no genetic abnormalities have been identified. ^[20]^

Whether there is relationship between genotype and phenotype in XLA patients remains to be delineated. Some studies support such correlation, whereas others demonstrated otherwise.[ ^15^ ^16^ ^20^] In this study, we found that there was no relationship between clinical symptoms and BTK mutations, but both the onset age and the diagnosis age of patients who had severe genotype were earlier than those who had mild genotype. This was similar to the results of the study by Lee et al that patients with less severe missense mutations had higher age of onset than those with severe missense mutations. ^[25]^ We reviewed these patients’ medical history, and found that the incidence of infection in patients with severe genotypes was higher than those with milder genotypes. We speculated that the doctors were more alert for the patients who often got infection, so the age of diagnosis was earlier.

There is no significant difference between the onset age of XLA in our research and that of other countries.[ ^11^ ^12^ ^14^ ^29^] The average diagnosis age in our research is 7.09 years, with 5 patients diagnosed within 1 year, 14 patients (8.05%) within 2 years, and 24 patients (13.79%) within 3 years. The average age of diagnosis of patients without family history is 5.37, with half within 2 years of age in the United States. ^[11]^ The average age of diagnosis is 6.2 years, with half diagnosed within 3 years of life in Spain ^[14]^ and 6.5 years in Holland. ^[29]^ Obviously, the age of diagnosis of XLA patients is rather late in mainland China.[ ^11^ ^14^] However, there has been significant improvement on this in XLA patients, with some patients being diagnosed at the stage of primary symptoms. Unfortunately, in the current study, all patients except patients 38 and 130 were not diagnosed at the time of primary symptoms. Since Chinese patients carry the same spectrum of *BTK* mutations as in other countries, the delayed age of diagnosis is probably due to the less awareness and low index of suspicion of community physicians. However, we noticed progress in recognition since many more cases were diagnosed after 2007, and the interval time between symptom onset and age of diagnosis was sharply decreased.

There are still some limitations of this study. First, we did not analyze the transcriptional and translational levels of *BTK* gene of these patients. This study is a retrospective study, so we did not carry out the analysis at the very beginning, and due to many reasons, a part of these patients could not be followed up. Furthermore, the advanced analysis of novel mutations, such as analysis of BTK by flow cytometry, immunofluorescence staining, and/or Western blotting, was not performed, which may weaken the comprehensive understanding of XLA patients.

## 5 Conclusions

We presented here the data of 174 patients with BTK mutations identified in our center over the past decade, which represents the largest number of XLA patients from China. A total of 127 mutations, including 45 previously unknown sequence variants, were detected. The spectrum of BTK mutations in China is similar to that of other countries and continents in BTK database.

The severe genotypes were associated with younger age of onset and positive family history. Other definite relationships between genotypes and phenotypes were not confirmed in this study. Further investigations need to be done to fully elucidate the genotype–phenotype correlation in XLA patients.

## Acknowledgments

We thank all the blood donors, Shanghai Jiao Tong University School of Medicine for assistance in blood collection, Hong Kong University for provision part of genetic testing, and Ms Rebecca Eisan for helping us edit the language.
